# Three-dimensional computed tomography evaluation of craniofacial characteristics according to lateral deviation of chin

**DOI:** 10.1186/s40902-019-0241-1

**Published:** 2019-12-06

**Authors:** Hyo-Won CHOI, Bola KIM, Jae-Young KIM, Jong-Ki HUH, Kwang-Ho PARK

**Affiliations:** Department of Oral and Maxillofacial Surgery, Gangnam Severance Hospital, Yonsei University College of Dentistry, Seoul, Korea

**Keywords:** Lateral deviation of chin, Upper and middle facial third asymmetry, Asymmetry of glenoid cavity

## Abstract

**Background:**

The relationship between the lateral deviation of chin and the upper and middle facial third asymmetry is still controversial. The purpose of this study is to evaluate the correlation of upper and middle facial third asymmetry with lateral deviation of chin using 3-dimensional computed tomography. The study was conducted on patients who underwent orthognathic surgery from January 2016 to August 2017. A total of 40 patients were included in this retrospective study. A spiral scanner was used to obtain the 3-dimensional computed tomography scans. The landmarks were assigned on the reconstructed 3-dimensional images, and their locations were verified on the axial, midsagittal, and coronal slices. The Pearson correlation analysis was performed to evaluate the correlation between chin deviation and difference between the measurements of distances in paired craniofacial structures. Statistical analysis was performed at a significance level of 5%.

**Results:**

In mandible, the degree of chin deviation was correlated with the mandibular length and mandibular body length. Mandibular length and mandibular body length are shorter on the deviated-chin side compared to that on the non-deviated side (mandibular length, *r =* −0.897, *p* value **<** 0.001; mandibular body length, *r =* −0.318, *p* value **=** 0.045). In the upper and middle facial thirds, the degree of chin deviation was correlated with the vertical asymmetry of the glenoid fossa and zygonion. Glenoid fossa and zygonion are superior on the deviated-chin side than on the non-deviated side (glenoid fossa, *r =* 0.317, *p* value **=** 0.046; zygonion, *r =* 0.357, *p* value **=** 0.024).

**Conclusion:**

Lateral deviation of chin is correlated with upper and middle facial third asymmetry as well as lower facial third asymmetry. As a result, treatment planning in patients with chin deviation should involve a careful evaluation of the asymmetry of the upper and middle facial thirds to ensure complete patient satisfaction.

## Background

Facial asymmetry is a relatively common feature with a prevalence rate of 21–85%. In majority of cases, facial asymmetry is mild and hardly recognizable, and hence, surgical intervention is not usually necessary [1–4]. However, patients with apparent facial asymmetry may not be satisfied with their appearance; such patients are more likely to opt for surgical intervention for esthetic and occlusal improvement [3].

The most common type of facial asymmetry is observed in the lower third of face with lateral deviation of the chin (75%) [3]. The most common cause is unilateral mandibular hyperplasia, i.e., enlargement of the mandible [5]. Functional disharmony of the masticatory muscles may be associated with lower facial third asymmetry with lateral deviation of chin [6].

Facial asymmetry often involves varying degrees of upper (5%) and middle (36%) facial third asymmetries [3]. In a previous study, asymmetry of the glenoid cavity, a type of upper and middle facial third asymmetry, was reported [7]. Asymmetry of the glenoid cavity is caused by defects in generation, proliferation, migration, and differentiation of cranial neural crest cells [8] or craniofacial structure modeling from the cerebrum [9–11]. As a result, asymmetry of the glenoid cavity causes lateral deviation of the chin [7].

The relationship between the lateral deviation of chin and the upper and middle facial third asymmetry is still controversial. López Buitrago et al. reported lateral deviation of chin is associated with upper and middle facial third asymmetry, while Kwon et al. reported lateral deviation of chin is not closely related with upper and middle facial third asymmetry [7, 12].The purpose of this study was to evaluate the correlation of upper and middle facial third asymmetry with lateral deviation of chin using 3-dimensional computed tomography (3-D CT).

## Methods

### Patients

The study was conducted on patients who underwent orthognathic surgery at the Department of Oral and Maxillofacial surgery, Gangnam Severance Hospital, Seoul, Korea, between January 2016 and August 2017. Patients with (1) history of trauma to the jaw and (2) congenital deformities, such as cleft lip and/or palate, were excluded from this study. Finally, 40 patients (18 males and 22 females; mean age, 25.50 years [range, 19 to 42]) were included this retrospective study. This study was approved by Gangnam Severance Hospital Institutional Review Board (Approval No. 3-2019-0119)

### Image acquisition and analysis

A spiral scanner was used for 3-D CT scans advised before orthognathic surgery for pre-surgical evaluation. (SOMATOM sensation 64; Siemens, Erlangen, Germany). During the process of CT scan, the patient’s teeth were maintained in centric occlusion, and the scan was obtained with following settings: gantry angle of 0°, 1024 × 1024 matrix, 120 kV, 90 mA, 1.0 mm slice thickness, and 0.5 sec gantry rotation time. The CT analysis software was used to reconstruct the digital imaging and communication in medicine (DICOM) images into 3-D images (Mimics version 23.0; Materalise Dental, Leuven, Belgium).

### 3-dimensional reference plane and craniofacial landmarks

**Fig. 1:**
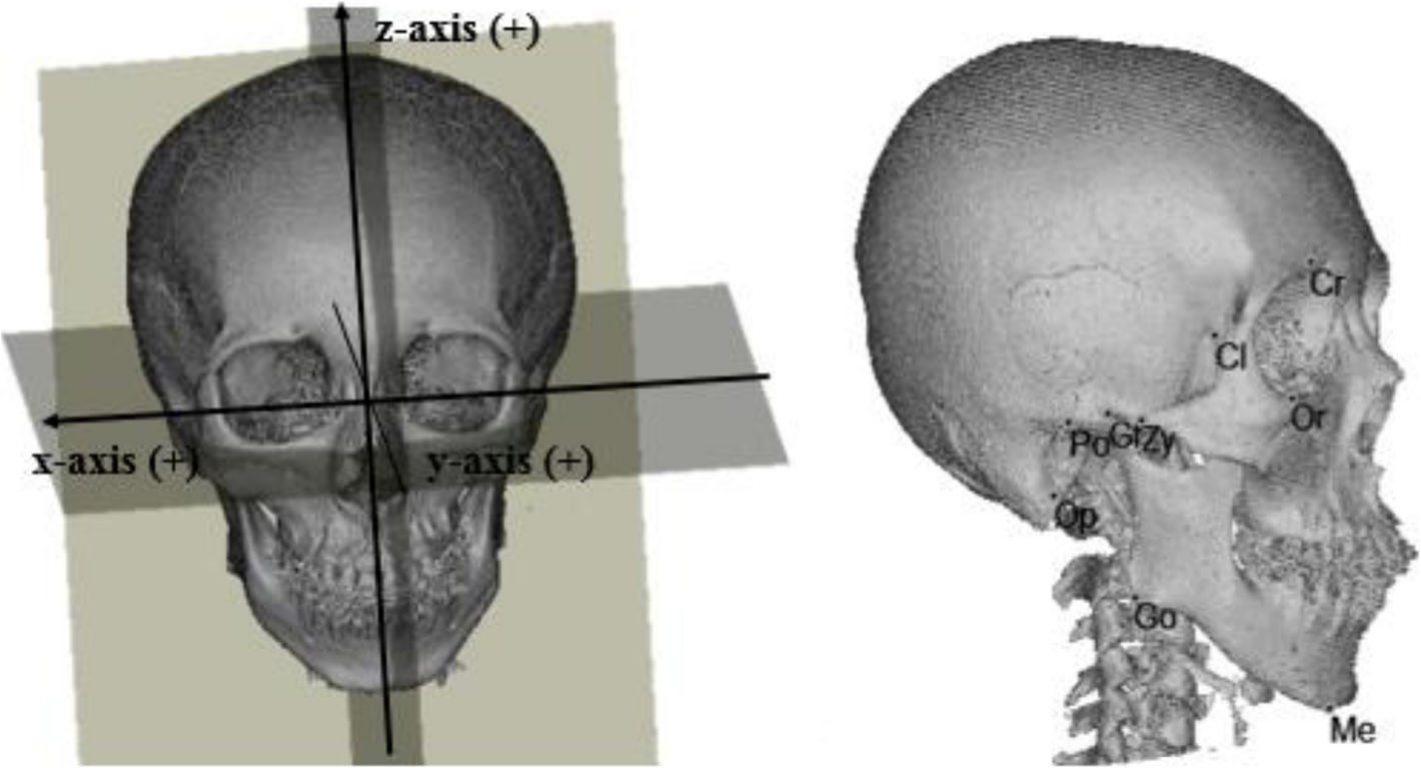
Three-dimensional reference planes and craniofacial landmarks. Cr, crista galli; Cl, clinoid process; Op, opisthion; Po, porion; Me, menton; Go, gonion; Gf, glenoid fossa; Or, orbitale; Zy, zygonion

**Fig. 2:**
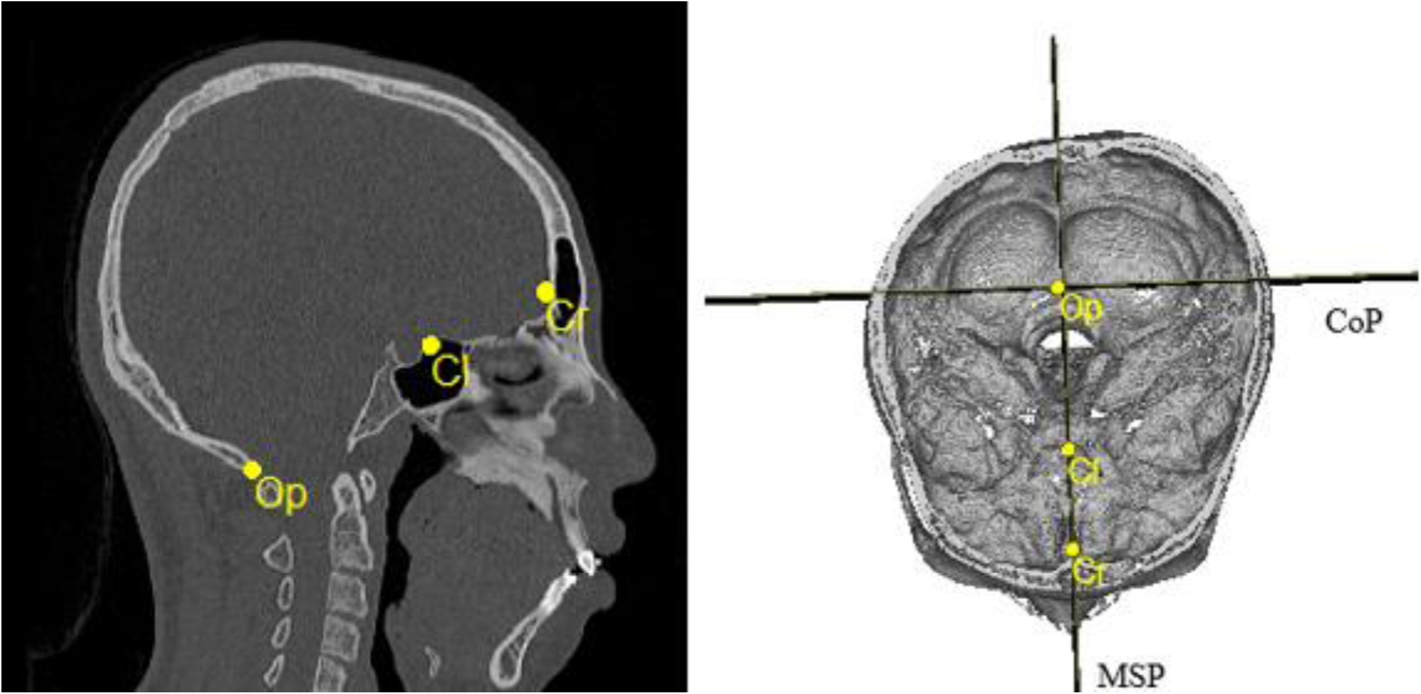
Computed tomography (CT) scans of craniofacial landmarks. Cr, crista galli; Cl, clinoid process; Op, opisthion; MSP, midsagittal plane; CoP, coronal plane

Landmarks were assigned on the reconstructed 3-D image, and their locations were verified on the axial, midsagittal, and coronal slices. The landmarks and measurements of the craniofacial structures to be performed were selected with reference to previous studies [12, 13]. The various landmarks studies are summarized in Figs. 1, 2 and Table 1.

**Table 1:** Description of craniofacial landmarks and reference planes

Point	Definition
Cr (crista galli)	The superior-most edge of the crista galli
Cl (clinoid process)	Midpoint between the anterior clinoid processes
Con (condylar superius)	The superior-most point of the condylar head; ConR: right, ConL: left
Op (opisthion)	Midpoint of the posterior arch of foramen magnum
Po (porion)	The superior-most point of the external auditory meatus; PoR: right, PoL: left
Me (menton)	The inferior-most point on the symphysis of mandible
Go (gonion)	The apex of the mandibular angle; GoR: right, GoL: left
Gf (glenoid fossa)	The antero-superior-most point of the glenoid fossa; GfR: right, GfL: left
Or (orbitale)	The inferior-most point of the infraorbital rim; OrR: right, OrL: left
Zy (zygonion)	The lateral-most point of the zygomatic arch; ZyR: right, ZyL: left
Axial plane (AxP)	A plane passing through PoR, PoL, and OrL
Midsagittal plane (MSP)	A plane perpendicular to the axial plane including Cr and Cl
Coronal plane (CoP)	A plane perpendicular to the axial plane and sagittal planes passing through Op

To determine the standard orientation, 3-D reference planes were initially located. The axial plane (AxP) was defined as a plane including the porion (Po) on both sides and the left orbitale (OrL). The midsagittal plane (MSP) was defined as a plane perpendicular to the AxP, including the crista galli (Cr) and the midpoint of the anterior clinoid process (Cl). The coronal plane (CoP) was defined as a plane perpendicular to the AxP and the MSP passing through opisthion (Op).

### Craniofacial measurements

The craniofacial measurements performed in the study are summarized in Table 2. Distance between the menton (Me) and MSP was defined as dMe, for convenience of comparison; (+) indicated right side deviation of menton. In the mandible, distance from the condylar superius (Con) to Me, distance from gonion (Go) to Me, and the distance from Con to Go were defined as mandibular length (dML), mandibular body length (dMBL), and ramal height (dRH), respectively. Distance from glenoid fossa (Gf) to MSP, CoP and AxP was defined as dGfx, dGfy, and dGfz, respectively. Distance from orbitale (Or) and zygonion (Zy) to each plane was defined as the same way.

**Table 2:** Description of craniofacial measurements of distances

	Definition	Description
dMe	Distance from Me to midsagittal plane	(+) indicates right side deviation of menton
dML	Distance from Con to Me	dMLR: right, dMLL: left
dMBL	Distance from Go to Me	dMBLR: right, dMBLL:left
dRH	Distance from Con to Go	dRHR: right, dRHL: left
dGfx	Distance from Gf to midsagittal plane	dGfxR: right, dGfxL: left
dOrx	Distance from Or to midsagittal plane	dOrxR: right, dOrxL: left
dZyx	Distance from Zy to midsagittal plane	dZyxR: right, dZyxL: left
dGfy	Distance from Gf to coronal plane	dGfyR: right, dGfyL: left
dOry	Distance from Or to coronal plane	dOryR: right, dOryL: left
dZyy	Distance from Zy to coronal plane	dZyyR: right, dZyyL: left
dGfz	Distance from Gf to axial plane	dGfzR: right, dGfzL: left
dOrz	Distance from Or to axial plane	dOrzR: right, dOrzL: left
dZyz	Distance from Zy to axial plane	dZyzR: right, dZyzL: left

*Abbreviations: *dML* refers to mandibular length; *dMBL* refers to mandibular body length; *dRH* refers to ramal height; *Me*, menton; *Con*, condylar superius; *Go*, gonion; *Gf*, glenoid fossa; *Or*, orbitale; *Zy*, zygonion

Differences between the measurements of distances in the paired craniofacial structures are given in Table 3. (R-L) was the mean difference between the measurements of distances in the paired craniofacial structures. (R-L) from MSP, CoP, and AxP was defined as x(R-L), y(R-L), and z(R-L). A positive value of x(R-L) indicates that the right craniofacial structure is more lateral than the left craniofacial structure from MSP, positive value of y(R-L) indicates that the right craniofacial structure is more anterior than the left craniofacial structure from CoP, and positive value of z(R-L) indicates that the right craniofacial structure is more superior than the left craniofacial structure from AxP. The relationship between the measurements of distances in the paired craniofacial structures and chin deviation was studied.

**Table 3:** Description of difference between the measurements of distances in paired craniofacial structures.

		Definition	Description
dML(R-L)		dMLR–dMLL	(R-L) refers to the difference between the measurements of distances in paired craniofacial structures (right–left)
dMBL(R-L)		dMBLR–dMBLL	
dRH(R-L)		dRHR–dRHL	
dGfx(R-L)		dGfxR–dGfxL	x(R-L) refers to the difference between the measurements of distances in paired craniofacial structures from MSP, and (+) indicates that the right craniofacial structure is more lateral than the left craniofacial structure from MSP
dZyx(R-L)		dZyxR–dZyxL	
dOrx(R-L)		dOrxR–dOrxL	
dGfy(R-L)		dGfyR–dGfyL	y(R-L) refers to the difference between the measurements of distances in paired craniofacial structures from CoP, and (+) indicates that the right craniofacial structure is more anterior than left craniofacial structure form CoP
dZyy(R-L)		dZyyR–dZyyL	
dOry(R-L)		dOryR–dOryL	
dGfz(R-L)		dGfzR–dGfzL	z(R-L) refers to the difference between the measurements of distances in paired craniofacial structures from AxP, and (+) indicates that the right craniofacial structure is more superior to the left craniofacial structure
dZyz(R-L)		dZyzR–dZyzL	
dOrz(R-L)		dOrzR–dOrzL	

*Abbreviations: (*R-L*), difference between measurements of distances in paired craniofacial structures (right–left); *dML* refers to mandibular length; *dMBL* refers to mandibular body length; *dRH* refers to ramal height; *dGfx*, distance from glenoid fossa to midsagittal plane; *dZyx*, distance from zygonion to midsagittal plane; *dOrx*, distance from orbitale to midsagittal plane; *dGfy*, distance from glenoid fossa to coronal plane; *dZyy*, distance from zygonion to coronal plane; *dOry*, distance from orbitale to coronal plane; *dGfz*, distance from glenoid fossa to axial plane; *dZyz*, distance from zygonion to axial plane; *dOrz*, distance from orbitale to axial plane

### Statistical analysis

To avoid inter-observer errors in measurements, all the measurements were performed by a single observer. The Pearson’s correlation analysis was performed to evaluate the correlation between chin deviation and difference between the measurements of distances in paired craniofacial structures. Statistical analysis was performed at a significance level of 5% with SPSS version 25.0 (IBM Corp, Armonk, NY, USA).

The intraclass correlation coefficient was used to evaluate intra-observer error by the same observer 1 week apart. In this study, the second set of measurements was used.

## Results

### Study subjects

The characteristics of patients included in the study are summarized in Table 4. A total of 40 patients (18 males and 22 females; mean age, 25.50 years [range, 19 to 42]) were included in this study. In our study, 18 patients (45%) showed chin deviation to the right side, and 22 patients (55%) showed chin deviation to the left side (mean, −1.82 mm [range, −16.44 mm to 8.44 mm]), and (+) indicates right side deviation of menton. Of the 40 patients, skeletal class III, class II, and class I malocclusions were evident in 33, 3, and 4 patients, as determined by lateral cephalograms.

**Table 4:** Patients characteristics (*N* = 40).

Characteristics	Categories	Number (percent)
Gender	Male	18 (45%)
	Female	22 (55%)
Age (years)	Mean	25.50
	Range	19 to 42
Chin deviation (direction)	Right	18 (45%)
	Left	22 (55%)
Chin deviation (mm)	Mean	−1.82
(+) indicates right side deviation of menton	Range	−16.44 to 8.44
Type of skeletal malocclusion	Skeletal class I malocclusion	4 (10%)
	Skeletal class II malocclusion	3 (8%)
	Skeletal class III malocclusion	33 (82%)

The intraclass correlation coefficients of craniofacial distance measurements are shown in Table 5. The intraclass correlation coefficient ranged from 0.91 to 0.99, which showed that data from one observer were very reliable.

**Table 5:** Intraclass correlation coefficient of craniofacial distance measurements (*N* = 40).

	Intraclass correlation coefficient (single)	95% confidence interval (single)
dMe	0.947	0.903–0.972
dML	dMLR: 0.994, dMLL: 0.994	dMLR: 0.988–0.997, dMLL: 0.988–0.997
dMBL	dMBLR: 0.987, dMBLL: 0.988	dMBLR: 0.976–0.993, dMBLL: 0.977–0.994
dRH	dRHR: 0.993, dRHL: 0.994	dRHR: 0.987–0.996, dRHL: 0.988–0.997
dGfx	dGfxR: 0.972, dGfxL: 0.965	dGfxR: 0.948–0.985, dGfxL: 0.934–0.981
dOrx	dOrxR: 0.971, dOrxL: 0.943	dOrxR: 0.945–0.984, dOrxL: 0.895–0.969
dZyx	dZyxR: 0.983, dZyxL: 0.941	dZyxR: 0.968–0.991, dZyxL: 0.891–0.968
dGfy	dGfyR: 0.973, dGfyL: 0.914	dGfyR: 0.949–0.986, dGfyL: 0.844–0.954
dOry	dOryR: 0.982, dOryL: 0.989	dOryR: 0.967–0.991, dOryL: 0.980–0.994
dZyy	dZyyR: 0.994, dZyyL: 0.987	dZyyR: 0.989–0.997, dZyyL: 0.976–0.993
dGfz	dGfzR: 0.982, dGfzL: 0.910	dGfzR: 0.966–0.990, dGfzL: 0.836–0.911
dOrz	dOrzR: 0.994	dOrzR: 0.988–0.997
dZyz	dZyzR: 0.933, dZyzL: 0.981	dZyzR: 0.876–0.964, dZyzL: 0.964–0.990

*Abbreviations: *dMe*, distance from Me to midsagittal plane; *dML*, mandibular length; *dMBL*, mandibular body length; *dRH*, ramal height; *dGfx*, distance from glenoid fossa to midsagittal plane; *dZyx*, distance from zygonion to midsagittal plane; *dOrx*, distance from orbitale to midsagittal plane; *dGfy*, distance from glenoid fossa to coronal plane; *dZyy*, distance from zygonion to coronal plane; *dOry*, distance from orbitale to coronal plane; *dGfz*, distance from glenoid fossa to axial plane; *dZyz*, distance from zygonion to axial plane; *dOrz*, distance from orbitale to axial plane

### The correlation between chin deviation and difference between the measurements of distances in paired lower facial third structures

The correlation between chin deviation and difference between the measurements of distances in paired lower facial third structures is shown in Table 6. In the lower facial thirds, the degree of chin deviation was related to mandibular length and mandibular body length. Mandibular length and mandibular body length are shorter on the deviated-chin side compared to that on the non-deviated side (mandibular length, *r =* − 0.897, value **<** 0.001; mandibular body length, *r =* − 0.318, *p* value **=** 0.045). However, no significant relation was observed between the degree of chin deviation and ramal height.

**Table 6:** The correlation between chin deviation and difference between the measurements of distances in paired lower facial third structures (*N* = 40).

	dMe		
	*R*	*p* value	
dML (R-L)	− 0.897	*< 0.001*	
dMBL (R-L)	− 0.318	*0.045*	
dRH (R-L)	− 0.123	0.449	

*Abbreviations: (*R-L*), difference between the measurements of distances in paired craniofacial structures (right–left); *dML* refers to mandibular length; *dMBL* refers to mandibular body length; *dRH* refers to ramal height

### The correlation between chin deviation and difference between the measurements of distances in paired upper and middle facial third structures

The correlation between chin deviation and difference between the measurements of distances in paired upper and middle facial third structures is shown in Table 7. In the upper and middle facial thirds, the degree of chin deviation was correlated with the vertical asymmetry of glenoid fossa and zygonion. Glenoid fossa and zygonion were superior on the deviated-chin side compared to that on the non-deviated side (glenoid fossa, *r =* 0.317, *p* value **=** 0.046; zygonion, *r =* 0.357, *p* value **=** 0.024). However, no significant relation was observed between the degree of chin deviation and position of orbitale.

**Table 7:** The correlation between chin deviation and difference between the measurements of distances in paired upper and middle facial third structures (*N* = 40).

	dMe		
	*R*	*p* value	
dGfx (R-L)	0.017	0.918	
dZyx (R-L)	0.310	0.051	
dOrx (R-L)	0.120	0.460	
dGfy (R-L)	0.018	0.914	
dZyy (R-L)	0.099	0.545	
dOry (R-L)	0.033	0.838	
dGfz (R-L)	0.317	*0.046*	
dZyz (R-L)	0.357	*0.024*	
dOrz (R-L)	0.189	0.242	

*Abbreviations: (*R-L*), difference between the measurements of distances in paired craniofacial structures (right–left); *dGfx*, distance from glenoid fossa to midsagittal plane; *dZyx*, distance from zygonion to midsagittal plane; *dOrx*, distance from orbitale to midsagittal plane; *dGfy*, distance from glenoid fossa to coronal plane; *dZyy*, distance from zygonion to coronal plane; *dOry*, distance from orbitale to coronal plane; *dGfz*, distance from glenoid fossa to axial plane; *dZyz*, distance from zygonion to axial plane; *dOrz*, distance from orbitale to axial plane

## Discussion

The purpose of this study was to evaluate the correlation between upper and middle facial third asymmetry and lateral deviation of chin using 3-D CT.

Traditionally, posteroanterior cephalograms, submentovertex view radiographs, or frontal facial photos have been used for diagnosing facial asymmetry. Certainly, these diagnostic modalities have proven their worth over the years. However, they have limited diagnostic abilities due to problems related to magnification, distortion, and superimposition of craniofacial structures [14–17]. However, 3-D CT reduces errors due to magnification and distortion and allows the quantitative measurements of craniofacial structures [18–20].

Currently, the external auditory meatus is regarded as a reliable reference for the analysis of craniofacial characteristics because of its stable shape [21]. Previous 3-D studies use the Frankfort’s horizontal plane as the reference axial plane [22–24]. For these reasons, in this study, the Frankfort’s horizontal plane passing through bilateral porion and left orbitale was used as the axial plane. Then, a plane perpendicular to the axial plane passing through the crita galli (Cr) and the midpoint between the anterior clinoid processes (Cl) was defined as midsagittal plane [12]. A plane perpendicular to axial and midsagittal plane with passing through opisthion (Op) was defined as a coronal plane based on the study of Kwon et al .[12].

The glenoid fossa is a depression in the temporal bone that articulates with the mandible to form the temporomandibular joint [25]. Positional changes in the glenoid fossa during growth can lead to facial asymmetry and malocclusion [7]. The location of the orbit and zygomatic bone plays an important role in facial symmetry and esthetics [26, 27]. For these reasons, the glenoid fossa, orbitale, and zygomatic arch were analyzed in this study. Mandibular length, mandibular body length, and ramal height were also analyzed to evaluate lower facial third asymmetry.

In the lower facial thirds, chin deviation is correlated with mandibular length and mandibular body length asymmetry, coincident with the findings of previous studies [12, 21, 28]. Moreover, in our study, chin deviation was also correlated with the upper and middle facial third asymmetry, especially vertical asymmetry of the glenoid fossa and zygomatic arch, coincident with the findings of another stud y[7]. In a previous study, asymmetry of the glenoid cavity, a type of upper and middle facial third asymmetry, was reported [7]. The asymmetry of glenoid cavity is often caused by the defects in generation, proliferation, migration, and differentiation of cranial neural crest cells [8] or craniofacial structure modeling from the cerebrum [9–11]. As a result, the glenoid cavity is located superiorly where developmental defects occurred (affected side) [7]. Similarly, supraorbital arch, zygomatic bone, and external auditory meatus are also located superiorly on affected side (orbiculo-zygomatic-meatal and articular asymmetry) [7]. Finally, the asymmetry of glenoid cavity functionally affects condylar position, causing lateral deviation of chin to the affected side [7].

This study showed that lateral deviation of chin is correlated with upper and middle facial third asymmetry as well as lower facial third asymmetry, especially vertical asymmetry of the glenoid fossa and zygomatic arch. Correction of chin deviation by mandibular surgery alone will not correct the asymmetry of the upper and middle facial thirds.

A limitation of this study is that a small number of craniofacial landmarks were analyzed for the correlation with the lateral deviation of chin, and further studies incorporating more number of craniofacial landmarks should be conducted for a deeper understanding of the correlation between the lateral deviation of chin and craniofacial landmarks.

To be best of our knowledge, this is the first study to evaluate craniofacial characteristics associated with the lateral deviation of chin using 3-dimensional imaging modalities. Considering the high prevalence and the impact of facial asymmetry on patient’s treatment outcome, this study is very relevant in the present scenario. Knowledge about the fact that facial symmetry is influenced by the upper and middle thirds of face will help clinicians around the world in proper treatment planning and hence, in providing better treatment to such patients.

## Conclusions

Lateral deviation of chin is correlated with upper and middle facial third asymmetry as well as lower facial third asymmetry. Correction of chin deviation by mandibular surgery alone will not correct the asymmetry of the upper and middle facial thirds. As a result, treatment planning in patients with chin deviation should involve a careful evaluation of the asymmetry of the upper and middle facial thirds to ensure complete patient satisfaction.

## Data Availability

The data sets used and/or analyzed during the current study are available from the corresponding author on reasonable request.

## Abbreviations

(R-L): Difference between the measurements of distances in paired craniofacial structures (right–left)
3-D CT: 3-dimensional computed tomography
AxP: Axial plane
Cl: Clinoid process
Con: Condylar superius
ConL: Left condylar superius
ConR: Right condylar superius
CoP: Coronal plane
Cr: Crista galli
dGfx: Distance from Gf to midsagittal plane
dGfx(R-L): dGfxR–dGfxL
dGfxL: Left dGfx
dGfxR: Right dGfx
dGfy: Distance from Gf to coronal plane
dGfy(R-L): dGfyR–dGfyL
dGfyL: Left dGfy
dGfyR: Right dGfy
dGfz: Distance from Gf to axial plane
dGfz(R-L): dGfzR–dGfzL
dGfzL: Left dGfz
dGfzR: Right dGfz
DICOM: Digital imaging and communication in medicine
dMBL: Mandibular body length, distance from Go to Me
dMBL(R-L): dMBLR–dMBLL
dMe: Distance from Me to midsagittal plane
dML: Mandibular length, distance from Con to Me
dML(R-L): dMLR–dMLL
dOrx: Distance from Or to midsagittal plane
dOrx(R-L): dOrxR–dOrxL
dOrxL: Left dOrx
dOrxR: Right dOrx
dOry: Distance from Or to coronal plane
dOry(R-L): dOryR–dOryL
dOryL: Left dOry
dOryR: Right dOry
dOrz: Distance from Or to axial plane
dOrz(R-L): dOrzR–dOrzL
dOrzL: Left dOrz
dOrzR: Right dOrz
dRH: Ramal height, distance from Con to Go
dRH(R-L): dRHR–dRHL
dZyx: Distance from Zy to midsagittal plane
dZyx(R-L): dZyxR–dZyxL
dZyxL: Left dZyx
dZyxR: Right dZyx
dZyy: Distance from Zy to coronal plane
dZyy(R-L): dZyyR–dZyyL
dZyyL: Left dZyy
dZyyR: Right dZyy
dZyz(R-L): dZyzR–dZyzL
dZyz: Distance from Zy to axial plane
dZyzL: Left dZyz
dZyzR: Right dZyz
Gf: Glenoid fossa
GfL: Left glenoid fossa
GfR: Right glenoid fossa
Go: Gonion
GoL: Left gonion
GoR: Right gonion
Me: Renton
MSP: Midsagittal plane
Op: Opithion
Or: Orbitale
OrL: Left orbitale
OrR: Right orbitale
Po: Porion
PoL: Left porion
PoR: Right porion
x(R-L): Difference between the measurements of distances in paired craniofacial structures from MSP
y(R-L): Difference between the measurements of distances in paired craniofacial structures from CoP
z(R-L): Difference between the measurements of distances in paired craniofacial structures from AxP
Zy: Zygonion
ZyL: Left zygonion
ZyR: Right zygonion
